# Investigation of Bis(Perfluoro‐*tert*‐Butoxy) Halogenates(I/III)

**DOI:** 10.1002/chem.202103325

**Published:** 2021-11-05

**Authors:** Patrick Pröhm, Willi R. Berg, Susanne M. Rupf, Patrick Voßnacker, Sebastian Riedel

**Affiliations:** ^1^ Department of Chemistry and Biochemistry-Inorganic Chemistry Freie Universität Berlin Fabeckstrasse 34/36 14195 Berlin Germany

**Keywords:** computational chemistry, fluorine chemistry, halogen chemistry, oxidation agents

## Abstract

A systematic study of halogenate(I/III) anions with polyatomic ligands is presented. The bis(perfluoro‐*tert*‐butoxy) halogenates(I) [X(OC_4_F_9_)_2_]^−^, X=Cl, Br, I, of chlorine, bromine, and iodine are prepared as their tetraethylammonium salts and characterized with IR, Raman, and NMR spectroscopic methods, as well as single‐crystal X‐ray diffraction analyses. Spectroscopical data are supported by quantum‐chemical calculations. Additionally, the bonding situation of the species in question are analyzed and discussed. Furthermore, the oxidation to the corresponding halogenate(III) derivatives was studied. For [Br(OC_4_F_9_)_2_]^−^, oxidation with elemental fluorine gave [BrF_2_(OC_4_F_9_)_2_]^−^. Iodide was directly oxidized by ClOC_4_F_9_ to the I^III^ species [I(OC_4_F_9_)_4_]^−^, which is a surprisingly inert anion that might be used as a weakly coordinating anion (WCA) in the future. For [Cl(OC_4_F_9_)_2_]^−^, the decomposition products of the synthetic approaches towards a chlorine(III) system were analyzed.

## Graphical abstract

**Chemistry with halogens** in positive oxidation states, especially the lighter halogens, is challenging due to their high oxidation potential. An ensemble of halogen +I and +III compounds with a comparatively large ligand system was synthesized and characterized with experimental and quantum‐chemical methods. This study is envisioned as an example for the diverse but sometimes overlooked chemistry of group 17.

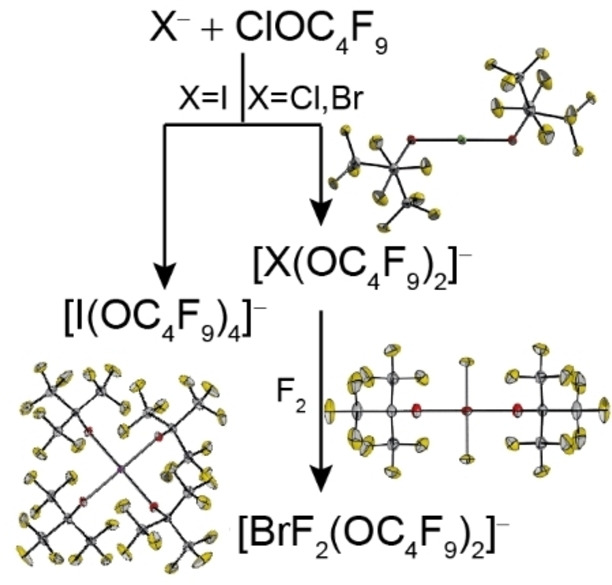

## Introduction

Halogen compounds with halogens in positive oxidation states and alcoholate ligands are rare, especially amongst the lighter halogens bromine[^1^, ^2^] and chlorine.[^3^, ^4^, ^5^] Partially positively charged halogen compounds with other ligands such as fluorido or oxido are more abundant, however in comparison to compounds with partially negatively charged halogens still rare.^[6]^ Despite their relatively low abundance they play an important role in modern synthetic chemistry. Hypervalent iodine compounds are widely used as reagents in organic and polymer chemistry.^[7]^ Bromine trifluoride can be used for a variety of bromination and fluorination reactions of organic molecules and its fluorido complex tetrafluoridobromate(III) is a promising tool for the recovery of scarce elements by urban mining.^[8]^ Hypochlorite is the main component of industrially used bleach^[9]^ and its organic esters are used for synthetical purposes, for example, the synthesis of perfluorinated peroxides.^[3]^ However, little is known about partially positively charged halogens with larger ligand systems. In case of multiatomic oxygen‐based ligands, organic hypohalites are the most common compounds. They are neutral compounds composed of an alcoholate fragment and a halogen in the oxidation state +I. Hypochlorites with different alcohol moieties are known, including OMe,^[4]^ OEt,^[4]^ O*i*Pr,^[5]^ O*t*Bu.^[5]^ Additionally, fully fluorinated versions of these hypochlorites exist.^[3]^ Generally, they are more reactive than their non‐fluorinated counterparts. While non‐fluorinated hypochlorites are typically prepared in aqueous solutions, their perfluorinated counterparts are synthesized with ClF under strict exclusion of moisture. The hypobromites BrOCF_3_ ^[1]^ and BrOC_4_F_9_ ^[2]^ are briefly described in the literature, however, they are significantly less stable than their chlorine counterparts.[^1^, ^2^] Examples for higher oxidation states than +I with multiatomic ligands are even more scarce. Neutral representatives include C_6_F_5_BrF_2_ and C_6_F_5_BrF_4_.^[10]^

Due to the high oxidation potential of partially positively charged halogens anions are often more stable than their neutral or cationic counterparts. Therefore, it appears surprising that virtually nothing is known about such anions with the exception of a quite instable intermediate reported by Minkwitz in 1997, [NMe_4_][Br(OCF_3_)_2_].^[11]^ It was synthesized via exposure of [NMe_4_]Br or [NMe_4_][BrCl_2_] to ClOCF_3_. However, decomposition of the anion under loss of carbonyl fluoride and formation of [BrF_2_]^−^ began already at −70 °C, a known problem of α‐fluoroalcohol moieties.^[11]^ Anions with polyatomic inorganic ligands such as OSO_2_F, ONO_2_, OClO_3_ and O_2_CCF_3_ are described in [NMe_4_][I(ONO_2_)_2_],^[12]^ [NMe_4_][I(ONO_2_)_4_],^[12]^ [NMe_4_][Br(ONO_2_)_2_],^[12]^ K[I(OSO_2_F)_4_],^[13]^ K[Br(OSO_2_F)_4_],^[13]^ Cs[I(OClO_3_)_4_],^[14]^ Cs[Br(OClO_3_)_2_]^[15]^ and Cs[I(O_2_CCF_3_)_4_],^[16]^ however, their characterization is often limited to elemental analysis and MIR spectra in case of ONO_2_ without the crucial region of the halogen oxygen vibrations. The only known crystal structure was recently reported by Seppelt for [NO_2_][Br(ONO_2_)_2_].^[17]^

**Figure 1:**
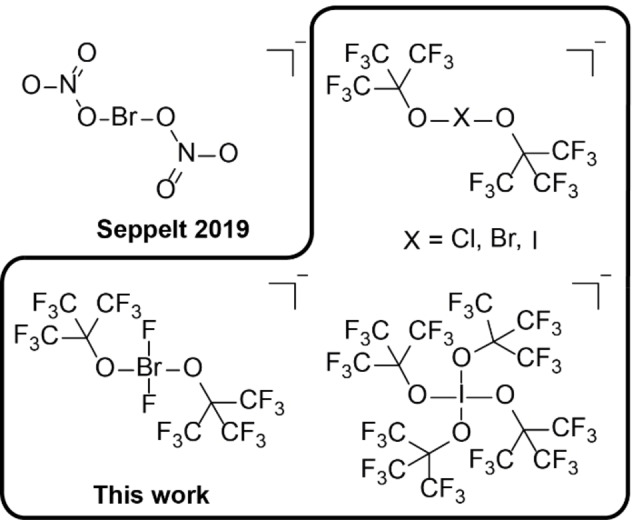
Comparison between the structurally characterized halogenate anions with polyatomic ligands.

Here we present the first systematic study of halogenate(I/III) anions of chlorine, bromine and iodine using the perfluorinated O*t*Bu^F^ as a potent ligand system to stabilize such oxidizing compounds (Figure 1). We believe this is a fundamental work for the understanding of hypervalent halogen molecules.

## Results and Discussion




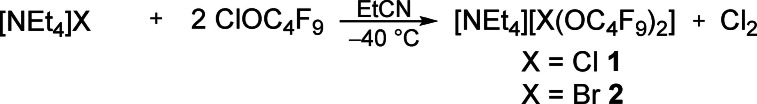




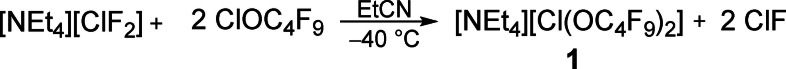




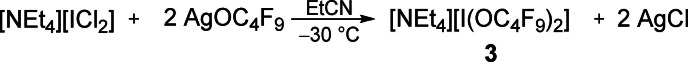




We were able to synthesize the halogenate(I) salts [NEt_4_][X(OC_4_F_9_)_2_], X=Cl, Br, I, for the three halogens chlorine, bromine and iodine. For chlorine and bromine the corresponding halide salts tetraethylammonium chloride or bromide were oxidized by perfluoro‐*tert*‐butylhypochlorite, ClOC_4_F_9_, in propionitrile at −40 °C (Eq. (1)) to yield [NEt_4_][Cl(OC_4_F_9_)_2_] (**1**) and [NEt_4_][Br(OC_4_F_9_)_2_] (**2**) under elimination of elemental chlorine. For **1**, [NEt_4_][ClF_2_] was also a suitable starting material (Eq. (2)). In this case no oxidation reaction of the halogen is necessary and the fluorido ligands are exchanged for perfluoro‐*tert*‐butoxy ligands under elimination of ClF. [NEt_4_][I(OC_4_F_9_)_2_] was obtained from the reaction of [NEt_4_][ICl_2_] with AgOC_4_F_9_ in propionitrile at −30 °C (Eq. (3)). Here, the ligand exchange was accomplished by precipitation of AgCl.

**Figure 2:**
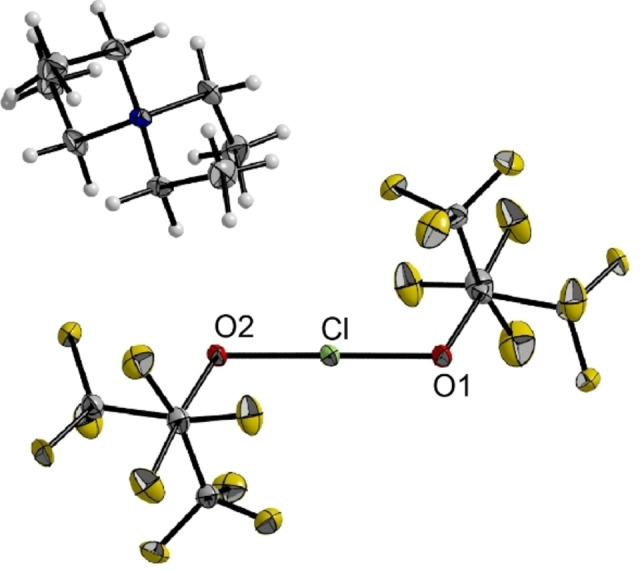
Molecular structure of [NEt_4_][Cl(OC_4_F_9_)_2_] **1**. Displacement ellipsoids are shown at 50 % probability level. Color code: yellow=fluorine, grey=carbon, red=oxygen, green=chlorine, blue=nitrogen. Compounds **2** and **3** are isotypic (cf. Supporting Information).

We obtained single crystals suitable for X‐ray diffraction for all three compounds by slowly cooling solutions in propionitrile (**1** and **2**) or DCM (**3**) to −80 °C. Interestingly, the compounds are all isotypic and crystallized in the monoclinic space group *P*2_1_ (see Figure 2 for **1**, for **2** and **3** see Figures S14 and S15). Table 1 gives an overview of the halogen oxygen bond lengths and angle in the three crystal structures. Overall, the anions are nearly inversion symmetric with bond angles close to 180° and nearly equally long X−O1 and X−O2 bond distances. As expected, the oxygen halogen bond length increases from chlorine to iodine due to the increasing atomic radii of the halogens. Comparing the oxygen halogen bond lengths with the very recently determined bond lengths from the crystal structures of [ClO]^−^ (168.6(1) pm) and [BrO]^−^ (182.0(3) pm) it becomes obvious that the bonding in the [X(OC_4_F_9_)_2_]^−^ anions is significantly weaker than the bonding in the hypohalite anions [XO]^−^.^[18]^ In case of **2**, a related structure is known, [NO_2_][Br(ONO_2_)_2_].^[17]^ Its Br−O bond lengths are well comparable (205.1(1) pm) and the O−Br−O angle is 180°, indicating a similar bonding situation. Additionally, we obtained Raman and IR data of compounds **1**–**3**. Overall, the spectra are very similar to each other. The Raman spectra (Figure S3) show the symmetric X−O stretching modes at 511 cm^−1^ (**1**), 513 cm^−1^ (**2**) and 513 cm^−1^ (**3**) (Table 2), with a slight blue shift when increasing the atomic number, whereas the X−O deformation mode experiences a red shift (459 cm^−1^ (**1**), 452 cm^−1^ (**2**), 431 cm^−1^ (**3**)). The IR spectra (Figure S6) are dominated by antisymmetric C−F valence vibrations in the area between 1300 and 1150 cm^−1^, followed by C−O valence and deformation modes between 1150 and 900 cm^−1^. Below 600 cm^−1^ several halogen oxygen modes as well as C−F deformation modes are observed. Like the symmetric X−O stretching modes, also the antisymmetric stretching modes experience a slight blue shift with increasing atomic number of the halogen from 505 cm^−1^ (**1**) over 507 cm^−1^ (**2**) to 510 cm^−1^ (**3**). The blue shifts indicate stronger bonding between the oxygen and halogen atoms when going to the heavier halogens. We confirmed this conclusion by calculating the energies of the decomposition reaction of [X(OC_4_F_9_)_2_]^−^ into XOC_4_F_9_ and [C_4_F_9_O]^−^ (X=Cl, Br, I). Indeed, we found that the decomposition reaction is the least endoenergetic for X=Cl, while for X=Br it is 30 kJ mol^−1^ more endoenergetic and for X=I even 57 kJ/mol. The stronger bonding for the heavier elements can be rationalized with stronger ionic contributions to the bonding. The natural population analysis (NPA) predicted a rise of the partial positive charge on the central halogen when going from chlorine to iodine (Cl=0.19, Br=0.31, I=0.44). This trend is easily understood when taking the decreasing electronegativity and ionization potential of the halogens into account. The charge of the halogens in the [X(OC_4_F_9_)_2_]^−^ series is very similar to the charges in the [XF_2_]^−^ series (Cl=0.23, Br=0.32, I=0.43). According to the natural bond orbital (NBO) analysis the bonding in the {O−X−O} core is a 3‐center‐4‐electron bond for all three molecules, similar to the fluoridohalogenates(I).

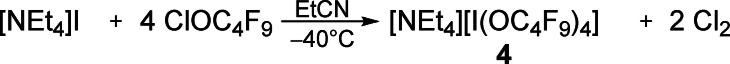




**Table 1:** Selected structural parameters of **1**, **2** and **3**, determined from their crystal structures in comparison with calculated parameters (italic). Bond lengths are given in [pm], bond angles in [°].

	X	X−O1	X−O2	O1−X−O2
**1**	Cl	195.6(2) *197.7*	195.0(2) *197.6*	179.4(1) *178.8*
**2**	Br	205.1(4) *209.3*	204.9(4) *209.1*	179.5(2) *179.7*
**3**	I	218.5(6) *221.7*	218.0(6) *221.7*	179.8(3) *179.6*

**Table 2:** Vibrational spectroscopic data of [NEt_4_][Cl(OC_4_F_9_)_2_] (**1**), [NEt_4_][Br(OC_4_F_9_)_2_] (**2**), [NEt_4_][I(OC_4_F_9_)_2_] (**3**), [NEt_4_][I(OC_4_F_9_)_4_] (**4**) and [NEt_4_][BrF_2_(OC_4_F_9_)_2_] (**5**). Calculated data were obtained on the B3LYP‐D3(BJ)/def2‐TZVPP level.

	ν_sy_(XO)		ν_as_(XO)		ν_sy_(BrF)		ν_as_(BrF)	
	Exp.	Calc.	Exp.	Calc.	Exp.	Calc.	Exp.	Calc.
**1**	511	504	505	499	–	–	–	–
**2**	513	504	507	500	–	–	–	–
**3**	513	503	510	500	–	–	–	–
**4**	519^[a]^ 541^[b]^	505^[a]^ 539^[b]^	510	506	–	–	–	–
**5**	521	509	515	504	477	473	515	502

[a] out‐of‐phase; [b] in‐phase.

**Figure 3:**
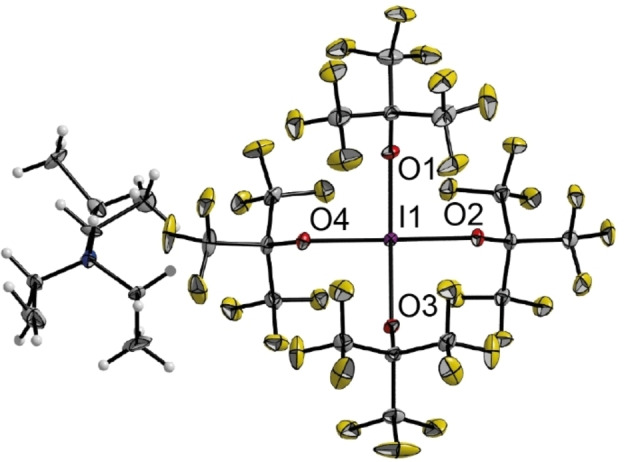
Molecular structure of [NEt_4_][I(OC_4_F_9_)_4_] **4**. One ion pair of the asymmetric unit is shown. Displacement ellipsoids are shown at 50 % probability level. Disorder in the cation omitted for clarity. Color code: yellow=fluorine, grey=carbon, red=oxygen, purple=iodine, blue=nitrogen. Selected bond lengths [pm] and angles [°]: I1−O1 213.1(2), I1−O2 211.7(2), I1−O3 212.4(2), I1−O4 212.7(2) O1−I1−O2 88.58(7), O2−I1−O3 90.85(7), O3−I1−O4 89.22(7), O4−I1−O1 91.34(7).

Due to the seemingly strong analogy between [X(OC_4_F_9_)_2_]^−^ and [XF_2_]^−^ we investigated the synthesis of the OC_4_F_9_ analogues of [XF_4_]^−^ anions with the central halogen in oxidation state +III. In contrast to the reaction of ClOC_4_F_9_ with Cl^−^ and Br^−^ which generated halogenate(I) compounds, the reaction of tetraethylammonium iodide [NEt_4_]I with four or more equivalents of ClOC_4_F_9_ under generation of chlorine yielded [NEt_4_][I(OC_4_F_9_)_4_] (**4**) with iodine in the desired oxidation state +III (Eq. (4)). Single crystals were obtained by slowly cooling a propionitrile solution to −80 °C. **4** crystallized in the monoclinic space group *P*2_1_/*n* with two ion pairs in the antisymmetric unit (Figure 3). The I−O bond lengths are in the range of 211.7(2) pm to 213.1(2) pm (calc: 214.7 pm) and therefore 5–7 pm shorter than in the I^I^ compound **3**. The bond angles are between 88.58(7)° (O1−I1−O2) and 91.34(7)° (O4−I1−O1). As for the halogenate(I) compounds, the IR spectrum (Figure S4) shows several prominent C−F valence vibrations in the area between 1300 cm^−1^ and 900 cm^−1^. The antisymmetric I−O valence vibrations are found at 511 cm^−1^, highly coupled with the C−F deformation modes in the ligand backbone. In the area below 400 cm^−1^ I−O deformation modes are observed, again coupled with the ligand. The Raman spectrum (Figure S7) shows the in‐phase symmetric I−O valence modes at 541 cm^−1^ and the out‐of‐phase mode at 519 cm^−1^. In comparison to the corresponding I^I^ species **3**, the symmetric I−O modes are slightly blue shifted (Δν_sy_=28 cm^−1^ for the in‐phase mode of **4**) indicating a stronger bonding in **4** than in **3**. Again, we calculated the decomposition reaction of [I(OC_4_F_9_)_4_]^−^ into I(OC_4_F_9_)_3_ and [C_4_F_9_O]^−^ and found that it is more endoenergetic by 57 kJ/mol than the respective decomposition of **3**. According to the NBO analysis the bonding situation is best described by two perpendicular 3‐center‐4‐electron bonds and the NPA predicted a charge of 1.7 for the iodine center.

In the area below 700 cm^−1^ a variety of valence and deformation modes of the {IO_4_} unit are observed. At higher energies (900 cm^−1^ to 1300 cm^−1^) weak bands corresponding to C−O and C−F vibrations are detected. We unsuccessfully tried to oxidize the iodine center further by adding stoichiometric and excess diluted fluorine. This is somewhat surprising because the addition of three equivalents of diluted fluorine (10 %) to a solution of [NEt_3_Me]I in propionitrile readily yields [NEt_3_Me][IF_6_] (see discussion in Supporting Information). Additionally, among the binary iodine fluorides the oxidation state +V is the most preferred one.^[19]^ However, the crystal structure of **4** depicted as a space‐filling model (Figure S12) reveals that the iodine center is sterically completely shielded by the ligands which is reminiscent of the aluminum derivative [Al(OC_4_F_9_)_4_]^−^ which is widely used as a WCA.^[20]^ The estimated thermochemical volume of **4** is 698 Å^3^ (see Supporting Information for details). This is similar to the volume of the aluminate [Al(OC_4_F_9_)_4_]^−^ (776 Å^3^).^[21]^ Additionally, we calculated the electrostatic potential (ESP) mapped on the electron density (Figure S13). It showed a well distributed negative charge without any concentrated charge spots. Therefore, we envision this anion to be a potent metal‐free WCA. Amongst the substances discussed in this work, [NEt_4_][I(OC_4_F_9_)_4_] is the most stable one. It can be stored at room temperature under Ar and for short amounts of time handled at ambient atmosphere (Figures S16 and S17). Hydrolysis under formation of I_2_ occurs within several minutes in comparison to immediate decomposition of the lighter homologues. Synthesis of a mixed ligand I^III^ anion from the reaction of [NEt_4_][ICl_2_] with two equivalents of ClOC_4_F_9_ yielded two anionic species, [ICl_4_]^−^ and [I(OC_4_F_9_)_4_]^−^, indicating dismutation of the ligands. Quantum‐chemical calculations confirm that the symmetric anions are indeed energetically more favorable than two equivalents of [ICl_2_(OC_4_F_9_)_2_]^−^.

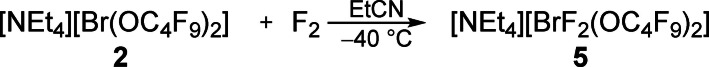




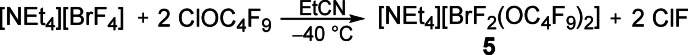




Additionally, we studied the reactivity of the lighter homologues **1** and **2**. The Br^I^ compound **2** can be oxidized with dilute fluorine (10 % in Ar) to yield the Br^III^ compound [NEt_4_][BrF_2_(OC_4_F_9_)_2_] **5** (Eq. (5)). The reaction is reminiscent of the fluorination of Cl^−^ and Br^−^ to the tetrafluorido halogenates(III) under similar conditions.[^22^, ^23^] Interestingly, we obtained the same compound from the ligand exchange reaction between [NEt_4_][BrF_4_] and ClOC_4_F_9_ under elimination of two equivalents of ClF (Eq. (6)).

**Figure 4:**
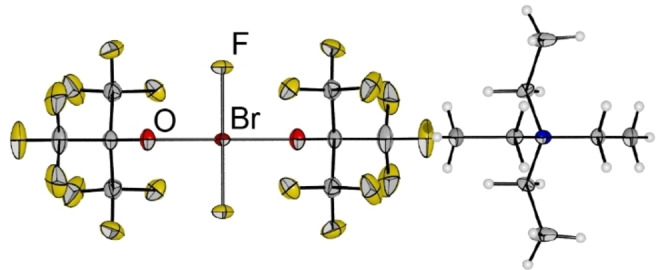
Molecular structure of [NEt_4_][BrF_2_(OC_4_F_9_)_2_] **5**. Displacement ellipsoids are shown at 50 % probability level. Disorder in the cation omitted for clarity. Color code: yellow=fluorine, grey=carbon, red=oxygen, dark red=bromine, blue=nitrogen. Selected bond lengths [pm]: Br−O 199.4(2), Br−F 189.3(2).

Again, we were able to characterize the compound by single crystal X‐ray diffraction (Figure 4). **5** crystallized in the monoclinic space group *I*2/*m*. The bromine center is positioned on a center of inversion, rendering the anion inversion symmetric. The Br−O distances are 199.4(2) pm (calc: 204.7 pm). The shortening by 5.5 ppm and 5.7 pm in comparison to **2** can be rationalized by the higher charge on the bromine leading to stronger ionic contributions in the bonding. The Br−F bonds have a length of 189.3(2) pm (calc: 190.1 pm). This is very similar to the Br−F bond lengths in [NEt_3_Me][BrF_4_] with 188.93(1) pm to 190.47(1) pm.^[23]^ Therefore we assume that the bonding situation in the anion of **2** is closely related to the bonding situation in [BrF_4_]^−^. This can also be deduced from the Raman spectrum (Figure S5). A breathing mode‐like vibration, i.e. ν_sy_(BrO) in‐phase coupled with ν_sy_(BrF), is observed at 521 cm^−1^ (Table 2). Additionally, the symmetric Br−F stretching mode is observed at 477 cm^−1^ as the dominant vibration in the spectrum. This is in excellent agreement with the a_1g_ and b_1g_ vibrations of [BrF_4_]^−^ at 519 cm^−1^ and 442 cm^−1^.^[23]^ In comparison with the Br^I^ species **2** the ν_sy_(BrO) mode is slightly blue shifted by 8 cm^−1^ due to the stronger bond formation which is in line with the structural data of the crystal structure. The NPA analysis shows a charge of +1.40 at the bromine which is significantly increased in comparison to **2** (+0.31), however, it is comparable to the natural charge of Br in [BrF_4_]^−^ (+1.46). Therefore, it can be assumed that the ionic contributions are increased in **5** in comparison to **2**. According to the NBO analysis the bonding of the {BrF_2_O_2_} core is best described by two perpendicular 3‐center‐4‐electron bonds. The IR spectrum shows both antisymmetric Br−F and Br−O stretching modes at 515 cm^−1^ (Table 2). In case of [NMe_4_][BrF_4_] the corresponding mode is observed at 480 cm^−1^.^[24]^ At lower energies the spectrum shows several wagging modes of the BrF_2_ unit, coupled with the ligand backbone, however, the most intense band at 288 cm^−1^ is in agreement with the out‐of‐plane vibration of [NMe_4_][BrF_4_] at 315 cm^−1^.^[24]^ The most intense IR bands (Figure S8) are observed between 900 and 1300 cm^−1^ and correspond to the ν_as_(CF) modes.

Analogous to the oxidation of **2** we performed a similar reaction with the Cl^I^ derivative **1**. However, in contrast to exclusive oxidation of the chlorine center we observed cleavage of the Cl−O bond. The reaction products were identified as the hypofluorite FOC_4_F_9_ and tetraethylammonium tetrafluoridochlorate(III) [NEt_4_][ClF_4_]. From the reaction of [NEt_4_][ClF_4_] with ClOC_4_F_9_ we obtained the bis(perfluoro‐*tert*‐butyl)peroxide (C_4_F_9_O)_2_.^[25]^ From these results we concluded that Cl^III^ is too strongly oxidizing to tolerate alcoholate ligands like OC_4_F_9_.

## Conclusion

The synthesis and characterization of halogenate anions [X(OC_4_F_9_)_2_]^−^ for the three halogens X=Cl, Br, and I in their oxidation state +I is presented. Their bonding situation was analyzed and differences in the bonding were elucidated showing more ionic contributions to the bonding for the heavier halogens. Additionally, their reactivity was studied by oxidizing the Br^I^ derivative with dilute fluorine. The resulting compound is a rare example of a Br^III^ anion with a polyatomic ligand system. At this point, sharp differences between the Br^I^ and the Cl^I^ compound manifested because fluorination of the latter led to a cleavage of the Cl−O bond instead of an oxidation of the halogen center.

In the case of iodine, the I^III^ compound [I(OC_4_F_9_)_4_]^−^ was obtained directly from the oxidation of iodide with ClOC_4_F_9_. The steric bulk of the perfluoroalcoholate ligands shielded the iodine center rendering it the most stable compound of the series (stable at RT and ambient atmosphere) and even showed resistance towards elemental fluorine. This anion is envisioned to be a potent, metal‐free candidate as a WCA for certain applications.

## Experimental Section

All experiments were performed under rigorous exclusion of moisture and oxygen using standard Schlenk techniques. Solids were handled in a dry box under argon atmosphere (O_2_<0.5 ppm, H_2_O<0.5 ppm). Fluorination experiments were performed with a stainless steel vacuum line, previously passivated with F_2_. Propionitrile was dried over Sicapent® prior to use and stored over molecular sieve 3 Å. [NEt_4_]Cl, [NEt_4_]Br and [NEt_4_]I were dried overnight at 120 °C under dynamic vacuum. All other chemicals were used as purchased. ClOC_4_F_9_ ^[26]^ and AgOC_4_F_9_ ^[27]^ were synthesized as described elsewhere. Raman spectra were recorded on a Bruker MultiRAM II equipped with a low‐temperature Ge detector (1064 nm, 30‐80 mW, resolution 4 cm^−1^). Spectra of single crystals were recorded at −196 °C using the Bruker RamanScope III. IR spectra were recorded on a Nicolet iS50 Advance FTIR by Thermo Fisher Scientific equipped with an ATR unit, with a Ge on KBr beamsplitter and a DLaTGS‐KBr detector for MIR and a solid‐substrate beamsplitter with a DLaTGS‐PE detector for FIR. For low‐temperature measurements we used a metal cylinder cooled by a cold N_2_ stream (see Figures S1 and S2 for details). NMR spectra were recorded on a JEOL 400 MHz ECS or ECZ spectrometer. All reported chemical shifts are referenced to the Ξ values given in IUPAC recommendations of 2008 using the ^2^H signal of the deuterated solvent as internal reference.^[28]^ For external locking acetone‐d6 was flame sealed in a glass capillary and the lock oscillator frequency was adjusted to give *δ*(^1^H)=7.26 ppm for a CHCl_3_ sample locked on the capillary. Crystal data were collected on a Bruker D8 Venture diffractometer with a Photon 100 CMOS area detector with MoKα radiation. Single crystals were picked at −80 °C under nitrogen atmosphere and mounted on a 0.15 mm Mitegen micromount using perfluoroether oil diluted with perfluorohexane. The structures were solved with the ShelXT^[29]^ structure solution program using intrinsic phasing and refined with the ShelXL^[30]^ refinement package using least squares minimizations by using OLEX2.4^[31]^ For visualization the Diamond V3.0 program was used.^[32]^

Deposition Numbers 2105613 (for **1**), 2105583 (for **2**), 2105590 (for **3**), 2105589 (for **4**), 2105582 (for **5**) and 2105587 (for **6**) contain the supplementary crystallographic data for this paper. These data are provided free of charge by the joint Cambridge Crystallographic Data Centre and Fachinformationszentrum Karlsruhe Access Structures service.

For structure optimizations and thermochemical data, the program package Turbomole V7.3^[33]^ was used with the DFT hybrid functional B3LYP^[34]^ and dispersion correction by Grimme (D3)^[35]^ and Becke‐Johnson damping (BJ)^[36]^ with the triple‐ζ basis set def2‐TZVPP^[37]^ and the effective core potential def2‐ECP^[38]^ for I. As a validation for minimum structures, harmonic frequencies were calculated as implemented. NBO analyses were performed with the Gaussian G16^[39]^ software package and NBO 7.0.^[40]^ Raman spectra were calculated with a 1064 nm excitation. Raman intensities are given for unpolarized radiation.

Synthesis of [NEt_4_][Cl(OC_4_F_9_)_2_] **1**: **Route A**: tetraethylammonium chloride (50.0 mg, 0.302 mmol, 1 eq) was dissolved in propionitrile (0.8 ml). Perfluoro‐*tert‐*butyl hypochlorite (324 mg, 1.20 mmol, 4 eq) was added at −196 °C. The reaction mixture was allowed to warm to −40 °C and stirred for 15 min. After slowly cooling to −80 °C the product was obtained as single crystals. **Route B**: A solution of [NEt_4_][ClF_2_] (61.5 mg, 0.302 mmol, 2 eq) in propionitrile (0.7 ml) was prepared as described elsewhere.^[22]^ Perfluoro‐*tert‐*butyl hypochlorite (163 mg, 0.603 mmol, 2 eq) was added at −196 °C. The reaction mixture was allowed to warm to −40 °C and stirred for 15 min. After slowly cooling to −80 °C the product was obtained as single crystals. ^1^H NMR (400 MHz, EtCN, ext. acetone‐d6, 223 K): *δ*/ppm=3.86 (q, ^3^ *J*(^1^H,^1^H)=7.6 Hz, 8H, CH_2_).^19^F NMR (376 MHz, EtCN, ext. acetone‐*d*6, 223 K): *δ*/ppm=−74.7. IR (ATR, 233 K) $\tilde{\nu }$ /cm^−1^=2996, 2961, 1490, 1457, 1445, 1397, 1369, 1236, 1212, 1176, 1154, 1105, 1055, 978, 959, 787, 764, 732, 723, 668, 578, 535, 505, 489, 366, 336, 318, 253, 174, 144. Raman (crystal, 1064 nm, 77 K)$\;\tilde{\nu }$ /cm^−1^=3019, 3005, 2996, 2954, 2900, 1468, 1301, 1102, 1003, 758, 679, 668, 576, 537, 527, 460, 422, 352, 338, 323, 294, 280, 212, 178, 117, 94, 73. CCDC number: 2105613.

Synthesis of [NEt_4_][Br(OC_4_F_9_)_2_] **2**: tetraethylammonium bromide (20.0 mg, 0.095 mmol, 1 eq) was dissolved in propionitrile (0.6 ml). Perfluoro‐*tert‐*butyl hypochlorite (154 mg, 0.570 mmol, 6 eq) was added at −196 °C. The reaction mixture was allowed to warm to −40 °C and stirred for 15 min. After slowly cooling to −80 °C the product was obtained as single crystals.^1^H NMR (400 MHz, CD_3_CN, 243 K): *δ*/ppm=2.33 (q, ^3^ *J*(^1^H,^1^H)=7.6 Hz, 8H, CH_2_), 1.13 (tt, ^3^ *J*(^1^H,^1^H)=7.6 Hz, ^3^ *J*(^14^N,^1^H)=1.9 Hz, 12H, CH_3_).^19^F NMR (376 MHz, CD_3_CN, 243 K): *δ*/ppm=−72.9 (s). IR (ATR, 233 K) $\tilde{\nu }$ /cm^−1^=2995, 2961, 1490, 1242, 1207, 1176, 1156, 1112, 1071, 1054, 1000, 976, 959, 788, 764, 730, 723, 671, 576, 535, 507, 475, 362, 335, 293, 288, 280, 222, 161, 131. Raman (crystal, 1064 nm, 77 K)$\;\tilde{\nu }$ /cm^−1^=3032, 2992, 2969, 2952, 1468, 1302, 1157, 1120, 999, 767, 676, 576, 536, 513, 452, 422, 322, 285, 267, 224, 213, 197, 117, 83. CCDC number: 2105583.

Synthesis of [NEt_4_][I(OC_4_F_9_)_2_] **3**: AgOC_4_F_9_ (131.0 mg, 0.381 mmol, 2.5 eq) was dissolved in EtCN (5 ml), cooled to −30 °C and added to a cooled (−30 °C) solution of tetraethylammonium dichloroiodate(I) (50.0 mg, 0.152 mmol, 1 eq) in EtCN (5 ml). The reaction mixture was stirred for 30 min at −30 °C, then for 30 min at RT. Then it was filtered and the solvent removed. The residual solid was washed with hexane three times (10 ml each). Single crystals were obtained by cooling a DCM solution to −80 °C after 1 night. IR (ATR, 298 K) $\tilde{\nu }$ /cm^−1^=3004, 1488, 1396, 1250, 1204, 1155, 1139, 1053, 1002, 785, 722, 676, 617, 565, 533, 510, 473, 453, 347, 305, 267, 219, 201,148. Raman (1064 nm, 298 K)$\;\tilde{\nu }$ /cm^−1^=3004, 2954, 2904, 1464, 1301, 1265, 1071, 1003, 973, 907, 894, 766, 679, 571, 536, 513, 421, 353, 335, 319, 293, 266, 214, 180, 150, 110. CCDC number: 2105590.

Synthesis of [NEt_4_][I(OC_4_F_9_)_4_] **4**: tetraethylammonium iodide (50.0 mg, 0.195 mmol, 1 eq) was dissolved in propionitrile (0.9 ml). Perfluoro‐*tert‐*butyl hypochlorite (262 mg, 0.972 mmol, 5 eq) was added at −196 °C. The reaction mixture was allowed to warm to RT and stirred for 15 min. All volatiles were removed under vacuum and the product was obtained as a colorless solid. ^1^H NMR (400 MHz, CD_3_CN, 298 K): *δ*/ppm=3.15 (q, ^3^ *J*(^1^H,^1^H)=7.3 Hz, 8 H, CH_2_), 1.20 (tt, ^3^ *J*(^1^H,^1^H)=7.3 Hz, ^3^ *J*(^14^N,^1^H)=1.9 Hz, 12 H, CH_3_). ^19^F NMR (376 MHz, CD_3_CN, 243 K): *δ*/ppm=−74.2 (s). IR (ATR, 233 K) $\tilde{\nu }$ /cm^−1^=3023, 3008, 1487, 1443, 1397, 1305, 1287, 1252, 1236, 1221, 1163, 1116, 1097, 999, 965, 783, 767, 725, 669, 573, 536, 510, 427, 357, 333, 319, 295, 274, 216, 187, 172, 151. Raman (1064 nm, 298 K)$\;\tilde{\nu }$ /cm^−1^=3006, 2969, 2952, 2895, 1467, 1354, 1291, 1227, 1155, 1117, 969, 772, 686, 575, 541, 519, 467, 451, 416, 366, 335, 322, 300, 281, 227, 191, 157, 110, 81. CCDC number: 2105589.

Synthesis of [NEt_4_][BrF_2_(OC_4_F_9_)_2_] **5**: **Route A**: **2** (160 mg, 0.238 mmol, 1 eq) was dissolved in propionitrile (3 ml) at −30 °C. Dilute fluorine (10 % in Ar) was bubbled through the solution for 3 min at a flow rate of 20 ml min^−1^ at −35 °C. After slowly cooling to −80 °C the product was obtained as single crystals. **Route B**: A solution of [NEt_4_][BrF_4_] (55 mg, 0.190 mol, 1 eq) in propionitrile (1 ml) was prepared as described elsewhere.^[19]^ Perfluoro‐*tert‐*butyl hypochlorite (308 mg, 1.14 mmol, 6 eq) was added at −196 °C. The reaction mixture was allowed to warm to −40 °C and stirred for 15 min. After slowly cooling to −80 °C the product was obtained as single crystals. IR (ATR, 233 K) $\tilde{\nu }$ /cm^−1^=3006, 1491, 1460, 1450, 1382, 1243, 1208, 1192, 1162, 1092, 1040, 1009, 996, 977, 964, 868, 808, 782, 767, 732, 724, 674, 578, 537, 515, 465, 358, 334, 323, 288, 248, 211, 186, 151, 118. Raman (crystal, 1064 nm, 77 K)$\;\tilde{\nu }$ /cm^−1^=3010, 2957, 1467, 996, 770, 734, 674, 538, 521, 477, 437, 416, 387, 326, 295, 192, 155, 131, 93, 76. CCDC number: 2105582.

## Conflict of interest

The authors declare no conflict of interest.

## Supporting information

As a service to our authors and readers, this journal provides supporting information supplied by the authors. Such materials are peer reviewed and may be re‐organized for online delivery, but are not copy‐edited or typeset. Technical support issues arising from supporting information (other than missing files) should be addressed to the authors.

Supporting Information
