# Supplementary material for: Investigation of Bis(Perfluoro‐tert‐Butoxy) Halogenates(I/III)
Source: Chemistry. 2021 Nov 5;27(70):17676–81. doi: 10.1002/chem.202103325 (PMC9298082; doi:10.1002/chem.202103325)
Supplement: Supplementary file 1 — Supporting Information [file CHEM-27-17676-s001.pdf]

Chemistry–A European Journal

Supporting Information

Investigation of Bis(Perfluoro-*tert*-Butoxy) Halogenates(I/III)

Patrick Pröhm, Willi R. Berg, Susanne M. Rupf, Patrick Voßnacker, and Sebastian Riedel*

Table of Contents

Low Temperature IR Setup 1
IR Data 2
Raman Data 4
[NEt_3Me][IF_6] 6
Crystal Data 7
Computational Data 11
References 15

Low Temperature IR Setup

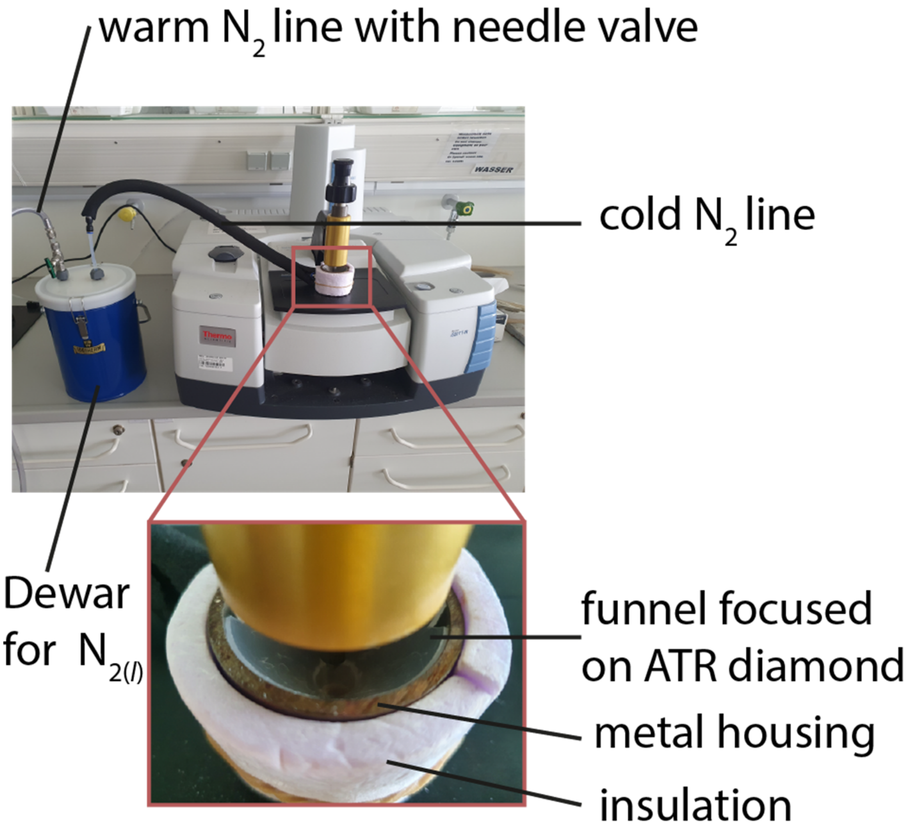

Figure S1. Experimental setup for measuring IR spectra at low temperatures.

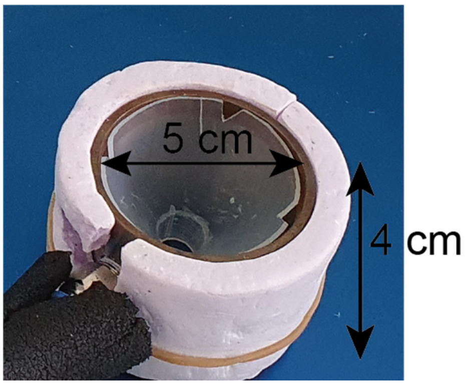

Figure S2. Experimental setup for measuring IR spectra at low temperatures.

Raman Data

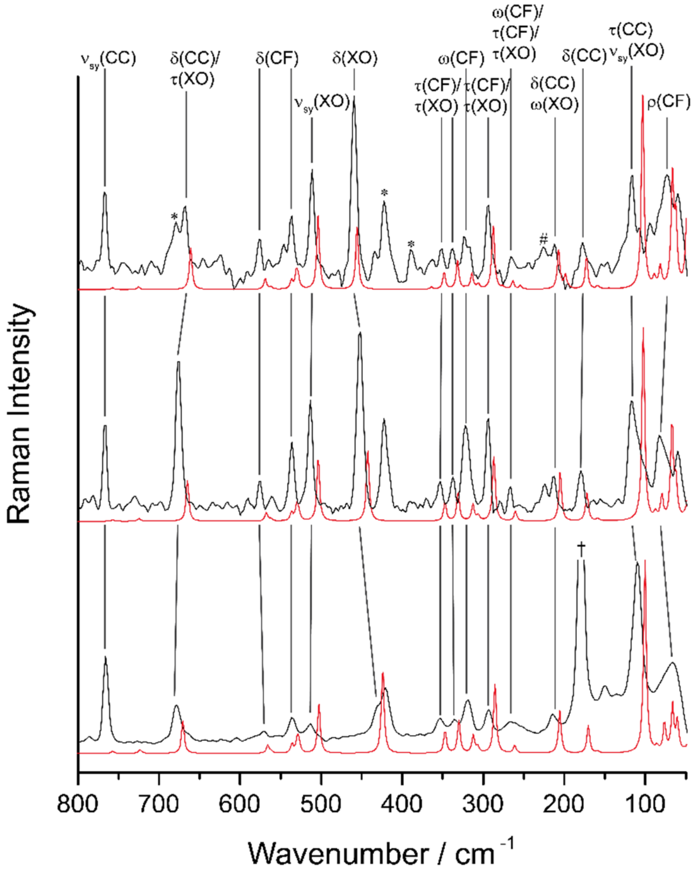

Figure S3. Raman spectra (1064 nm) of $[NEt_4][Cl(OC_4F_9)_2]$ **1** (top, 77 K), $[NEt_4][Br(OC_4F_9)_2]$ (middle, 77 K) and $[NEt_4][I(OC_4F_9)_2]$ (bottom, 298 K). Experimental spectra (black), calculated spectra of the anion (red, B3LYP-D3BJ/def2-TZVPP). Asterisk highlights cation bands, hash denotes residual solvent bands of EtCN, dagger highlights iodine impurity.

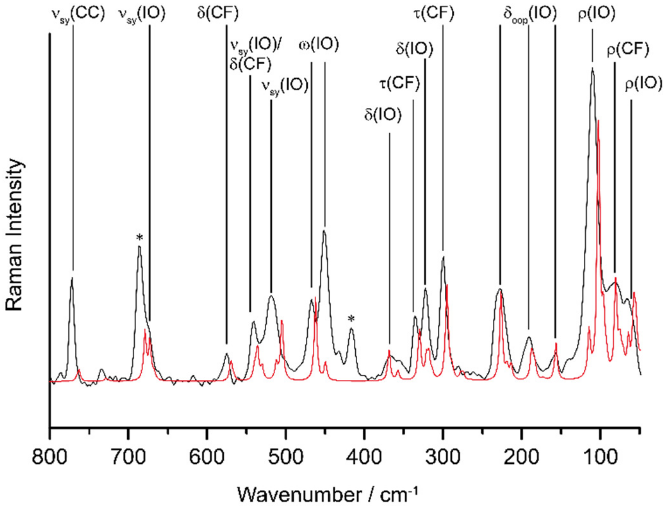

Figure S4. Raman spectrum (1064 nm, 298 K) of $[NEt_4][I(OC_4F_9)_4]$. Experimental spectrum (black), calculated spectrum of the anion (red, B3LYP-D3BJ/def2-TZVPP). Asterisk highlights cation bands.

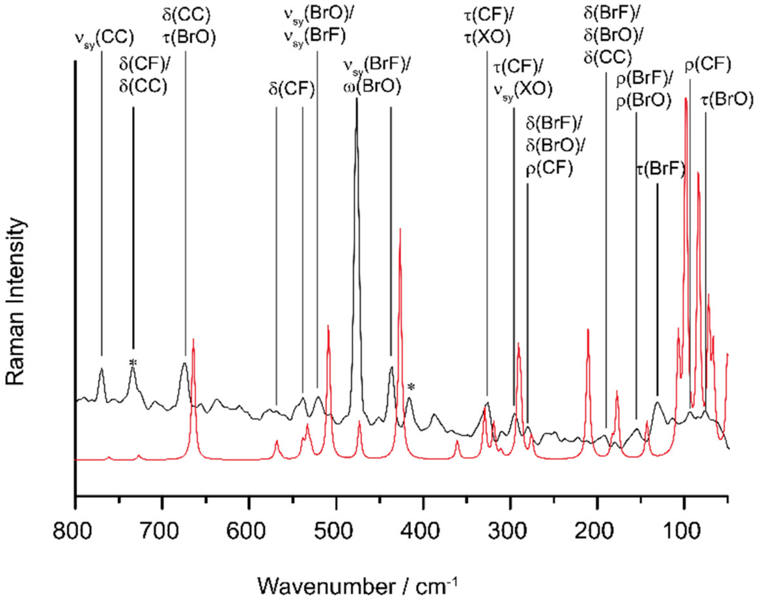

Figure S5. Raman spectrum (1064 nm, 77 K) of $[NEt_4][BrF_2(OC_4F_9)_2]$. Experimental spectrum (black), calculated spectrum of the anion (red, B3LYP-D3BJ/def2-TZVPP). Asterisk highlights cation bands.

IR Data

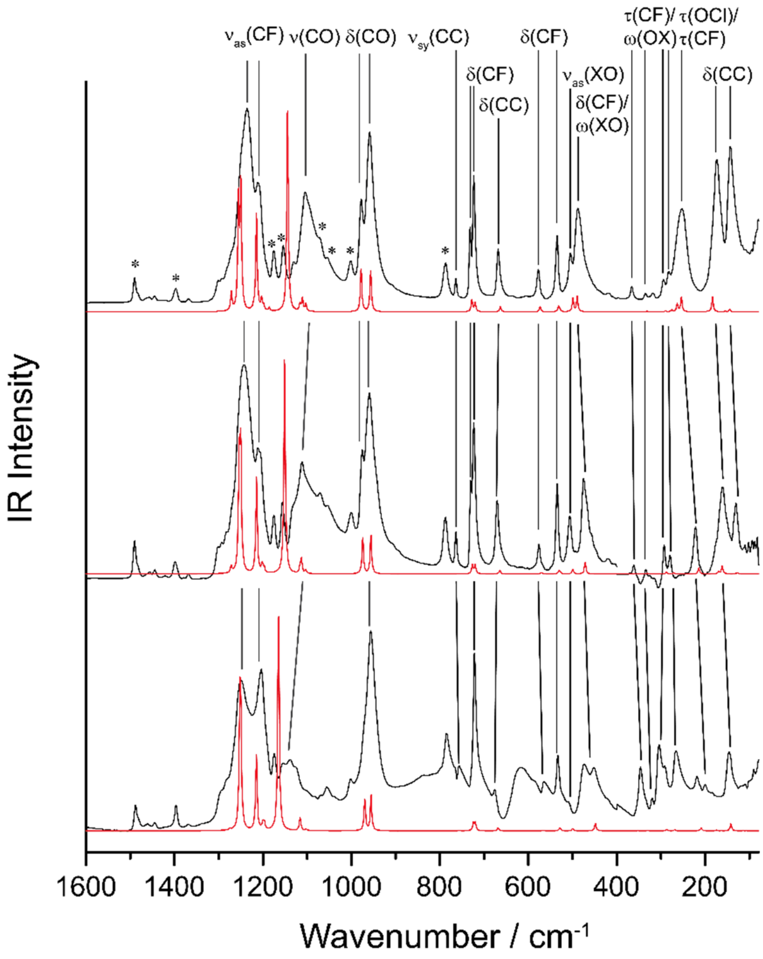

Figure S6. IR spectra (ATR) of [NEt_4][$Cl(OC_4F_9)_2$] (top, 233 K), [NEt_4][$Br(OC_4F_9)_2$] (middle 233 K) and [NEt_4][$I(OC_4F_9)_2$] (bottom, 298 K). Experimental spectra (black), calculated spectra of the anion (red). Discontinuity at 400 cm^{-1} due to a different beam splitter. Bands highlighted with an asterisk correspond to the cation.

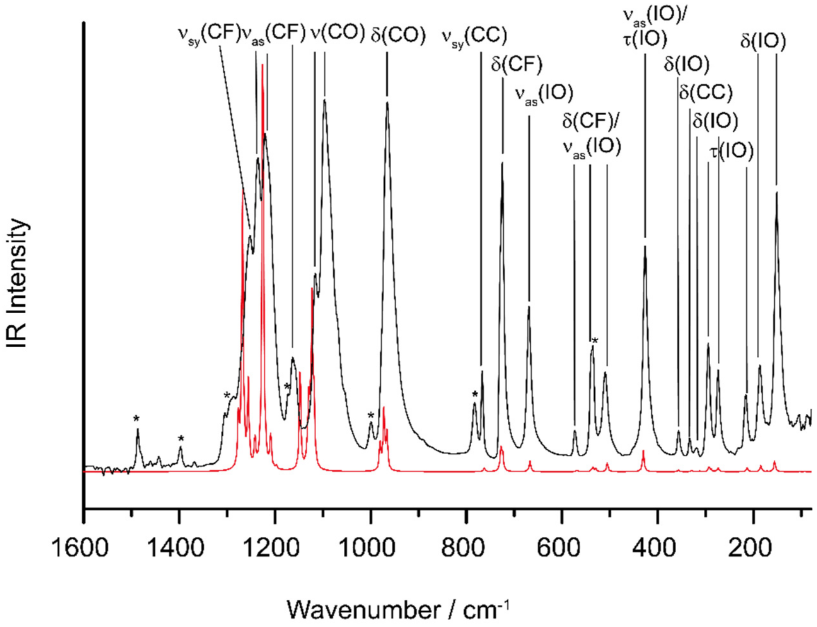

Figure S7. IR spectrum (ATR, 233 K) of [NEt_4][$I(OC_4F_9)_4$]. Experimental spectrum (black), calculated spectrum of the anion (red). Discontinuity at 400 cm^{-1} due to a different beam splitter. Bands highlighted with an asterisk correspond to the cation.

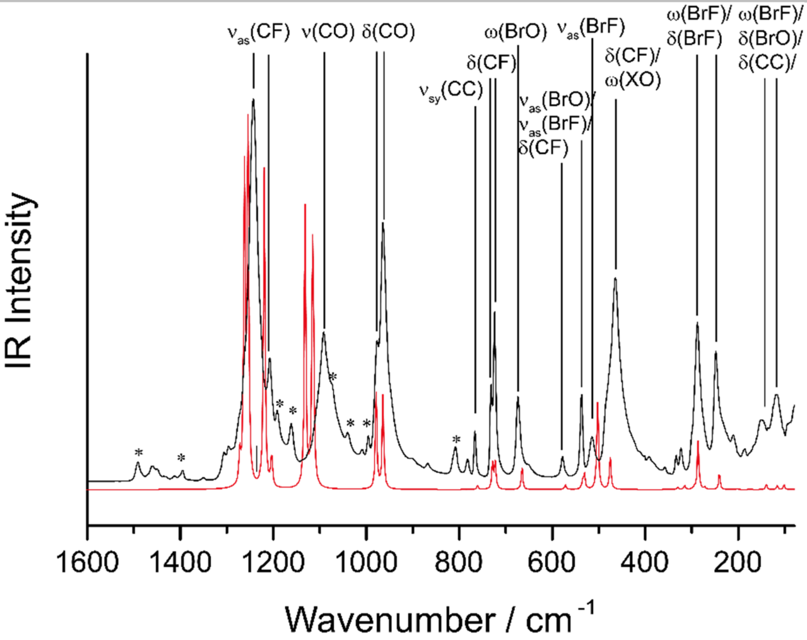

Figure S8. IR spectrum (ATR, 233 K) of $[NEt_4][BrF_2(OC_4F_9)_2]$. Experimental spectrum (black), calculated spectrum of the anion (red). Discontinuity at 400 cm^{-1} due to a different beam splitter. Bands highlighted with an asterisk correspond to the cation.

[NEt_3Me][IF_6] 6

Analogous to the reactions of quaternary tetraalkylammonium chlorides[1] and bromides[2] the iodide salt [NEt_3Me]I can be fluorinated using dilute fluorine (10 % in Ar) to yield the corresponding hexafluoridoiodate(V) salt (eq. 1).

$$[NEt_3Me]I \quad + \quad 3\,F_2 \xrightarrow[-40\ ^\circ C]{EtCN} [NEt_3Me][IF_6] \qquad (1)$$

We were able to obtain single crystals by cooling the reaction mixture slowly to −80 °C (Figure S7 and S8). As expected, the structure is largely in agreement with the known structure of [NMe_4][IF_6] which was synthesized from [NMe_4]F and IF_5.[3]

[NEt_3Me]I (100 mg, 0.411 mmol, 1 eq) was dissolved in propionitrile (2 ml) and cooled to −40 °C. Dilute fluorine (10 % in Ar, 20 ml min^{-1}, 15 min, 1 eq) was bubbled through the reaction solution. Afterwards, Ar was bubbled through the solution for 15 min to remove any residual reactive gas. The reaction mixture was slowly cooled to −80 °C and the product was obtained as a crystalline solid.
Raman (crystal, 1064 nm, 77 K) $\tilde{\nu}$ / cm^{-1} = 3050, 3000, 2946, 1507, 1077, 1008, 961, 683, 628, 565, 496, 460, 386.
CCDC number: 2105587

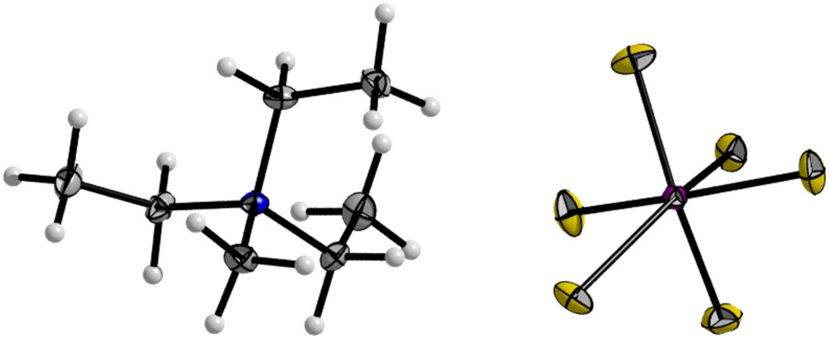

Figure S9. Solid-state structure of [NEt_3Me][IF_6]. Displacement ellipsoids are shown at 50 % probability level. Color code: yellow = fluorine, grey = carbon, purple = iodine, blue = nitrogen.

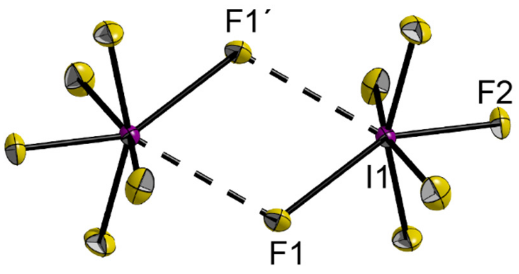

Figure S10. Solid-state structure of [NEt_3Me][IF_6] showing two bridged anions. Cations omitted for clarity. Displacement ellipsoids are shown at 50 % probability level. Color code: yellow = fluorine, purple = iodine. Selected bond lengths [pm]: I1-F1 222.4(2), I1-F2 186.6(2), I1-F1’ 269.0(2).

SUPPORTING INFORMATION

Crystal Data

Compound	**1**	**2**	**3**
Identification code	fullp21m_b	P21_twin_final	P2(1)_multiscan
Empirical formula	$C_{16}H_{20}ClF_{18}NO_2$	$C_{16}H_{20}BrF_{18}NO_2$	$C_{16}H_{20}F_{18}INO_2$
Formula weight	635.78	680.24	727.23
Temperature/K	100	100.0	100
Crystal system	monoclinic	monoclinic	monoclinic
Space group	$P2_1$	$P2_1$	$P2_1$
a/Å	9.8030(5)	9.9124(5)	10.1009(6)
b/Å	12.2085(7)	12.0667(6)	11.8498(7)
c/Å	10.5701(4)	10.5647(6)	10.7012(5)
α/°	90	90	90
β/°	113.137(2)	112.206(2)	111.355(2)
γ/°	90	90	90
Volume/Å^3	1163.28(10)	1169.92(11)	1192.92(12)
Z	2	2	2
ρ_{calc} g/cm^3	1.815	1.931	2.025
μ/mm^{-1}	0.324	1.916	1.499
F(000)	636.0	672.0	708.0
Crystal size/mm^3	0.938 × 0.521 × 0.517	0.59 × 0.23 × 0.19	0.353 × 0.167 × 0.143
Radiation	MoK_α (λ = 0.71073)	MoK_α (λ = 0.71073)	MoK_α (λ = 0.71073)
2Θ range for data collection/°	4.19 to 56.65	4.164 to 56.612	4.086 to 50.716
Index ranges	-13 ≤ h ≤ 13, -16 ≤ k ≤ 16, -14 ≤ l ≤ 13	-13 ≤ h ≤ 13, -16 ≤ k ≤ 16, -14 ≤ l ≤ 14	-12 ≤ h ≤ 12, -14 ≤ k ≤ 14, -12 ≤ l ≤ 11
Reflections collected	53521	5805	20495
Independent reflections	5773 [R_{int} = 0.0329, R_{sigma} = 0.0182]	5805 [R_{int} = 0.0, R_{sigma} = 0.0309]	4350 [R_{int} = 0.0553, R_{sigma} = 0.0411]
Data/restraints/parameters	5773/1/348	5805/1/348	4350/1/336
Goodness-of-fit on F^2	1.051	1.019	1.039
Final *R* indexes [I>=2σ (I)]	R_1 = 0.0382, wR_2 = 0.1032	R_1 = 0.0381, wR_2 = 0.0886	R_1 = 0.0410, wR_2 = 0.1010
Final *R* indexes [all data]	R_1 = 0.0407, wR_2 = 0.1049	R_1 = 0.0443, wR_2 = 0.0925	R_1 = 0.0510, wR_2 = 0.1075
Largest diff. peak/hole / e Å^{-3}	0.79/-0.29	0.65/-0.58	1.91/-1.02
Flack parameter	0.41(9)	0.481(15)	0.33(5)

Compound	**4**	**5**	**6**
Identification code	P21_n	I2_m	P21_n
Empirical formula	$C_{24}H_{20}F_{36}INO_4$	$C_{16}H_{20}BrF_{20}NO_2$	$C_7H_{18}F_6IN$
Formula weight	1197.31	718.24	357.12
Temperature/K	100	100.0	100.0
Crystal system	monoclinic	monoclinic	monoclinic
Space group	$P2_1/n$	$I2/m$	$P2_1/n$
a/Å	19.0494(7)	18.399(2)	8.3739(4)
b/Å	18.9420(5)	9.2277(9)	16.3141(8)
c/Å	20.5628(7)	6.9961(7)	9.6506(5)
α/°	90	90	90
β/°	96.6270(10)	95.600(4)	112.395(2)
γ/°	90	90	90
Volume/Å^3	7370.2(4)	1182.1(2)	1218.96(11)
Z	8	2	4
ρ_{calc} g/cm^3	2.158	2.018	1.946
μ/mm^{-1}	1.088	1.914	2.675
F(000)	4640.0	708.0	696.0
Crystal size/mm^3	0.26 × 0.22 × 0.17	0.37 × 0.33 × 0.3	0.2 × 0.2 × 0.2
Radiation	MoK$_\alpha$ (λ = 0.71073)	MoK$_\alpha$ (λ = 0.71073)	MoK$_\alpha$ (λ = 0.71073)
2Θ range for data collection/°	4.532 to 52.764	4.448 to 56.706	4.994 to 56.636
Index ranges	-23 ≤ h ≤ 23, -23 ≤ k ≤ 23, -25 ≤ l ≤ 25	-24 ≤ h ≤ 24, -12 ≤ k ≤ 12, -9 ≤ l ≤ 9	-11 ≤ h ≤ 10, -21 ≤ k ≤ 21, -12 ≤ l ≤ 12
Reflections collected	141718	12186	27477
Independent reflections	15060 [R_{int} = 0.0524, R_{sigma} = 0.0270]	1561 [R_{int} = 0.0348, R_{sigma} = 0.0213]	3029 [R_{int} = 0.0255, R_{sigma} = 0.0132]
Data/restraints/parameters	15060/0/1318	1561/0/116	3029/0/140
Goodness-of-fit on F^2	1.006	1.159	1.217
Final R indexes [I>=2σ (I)]	R_1 = 0.0319, wR_2 = 0.0714	R_1 = 0.0268, wR_2 = 0.0683	R_1 = 0.0190, wR_2 = 0.0461
Final R indexes [all data]	R_1 = 0.0476, wR_2 = 0.0778	R_1 = 0.0268, wR_2 = 0.0683	R_1 = 0.0191, wR_2 = 0.0462
Largest diff. peak/hole / e Å^{-3}	0.81/-0.61	0.42/-0.44	0.35/-1.28
Flack parameter	-	-	-

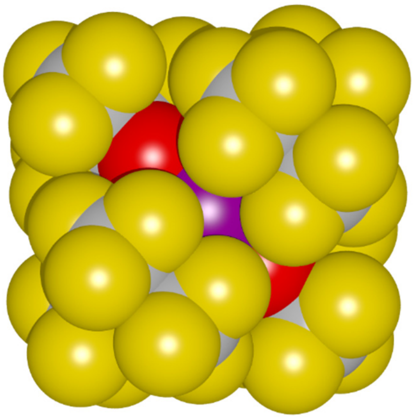

Figure S12. Space-filling representation of the anionic moiety of $[NEt_4][I(OC_4F_9)_4]$ **4**, taken from its solid-state structure. Color code: yellow = fluorine, red = oxygen, purple = iodine.

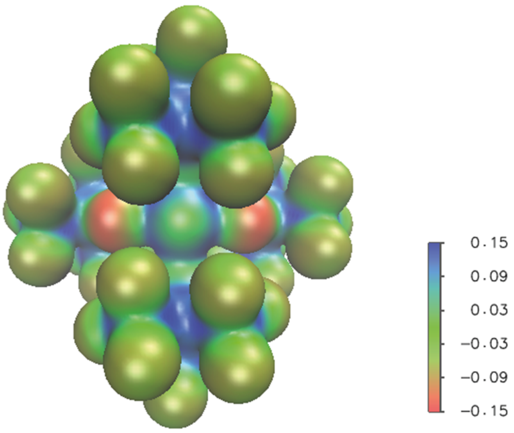

Figure S13. Electrostatic potential plotted in the electron density isosurface (iso = 0.025) of $[I(OC_4F_9)_4]^-$.

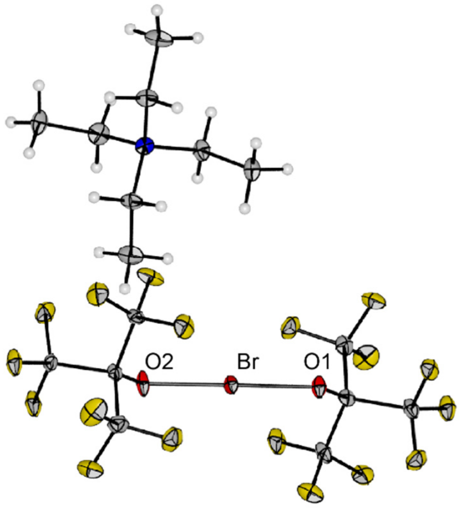

Figure S14. Molecular structure of $[NEt_4][Br(OC_4F_9)_2]$ **2**. Displacement ellipsoids are shown at 50 % probability level. Color code: yellow = fluorine, grey = carbon, red = oxygen, dark red = bromine, blue = nitrogen. Selected bond lengths [pm] and angles [°]: Br-O1 205.1(4), Br-O2 204.9(4); O1-Br-O2-179.5(2).

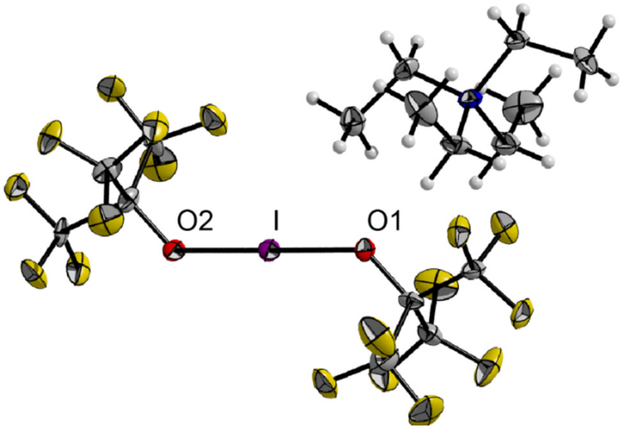

Figure S15. Molecular structure of $[NEt_4][I(OC_4F_9)_2]$ **3**. Displacement ellipsoids are shown at 50 % probability level. Disorder in the cation omitted for clarity. Color code: yellow = fluorine, grey = carbon, red = oxygen, purple = iodine, blue = nitrogen. Selected bond lengths [pm] and angles [°]: I-O1 218.5(6), I-O2 218.0(6), O1-I-O2 179.8(3).

The estimation of the thermochemical volume V_{therm} of the $[I(OC_4F_9)_4]^-$ anion from the crystal structure was done according to a method presented by Passmore.[4] Firstly, the thermochemical volume of the counterion $[NEt_4]^+$ was estimated from the crystal structure of $[NEt_4]Cl$[5].

$$\boldsymbol{V([\mathrm{NEt_4}]^+)_{therm} = \frac{unit\ cell\ volume}{Z} - V(\mathrm{Cl}^-) = \frac{990.7}{4} - 25 = 223\ \text{Å}^3}$$

With this the thermochemical volume V_{therm} of the $[I(OC_4F_9)_4]^-$ anion was estimated to 699 Å^3.

$$V([\mathrm{I(OC_4F_9)_4}]^-)_{therm} = \frac{unit\ cell\ volume}{Z} - \boldsymbol{V}([\mathrm{NEt_4}]^+) = \frac{7370.2}{8} - 223 = 698\ \text{Å}^3$$

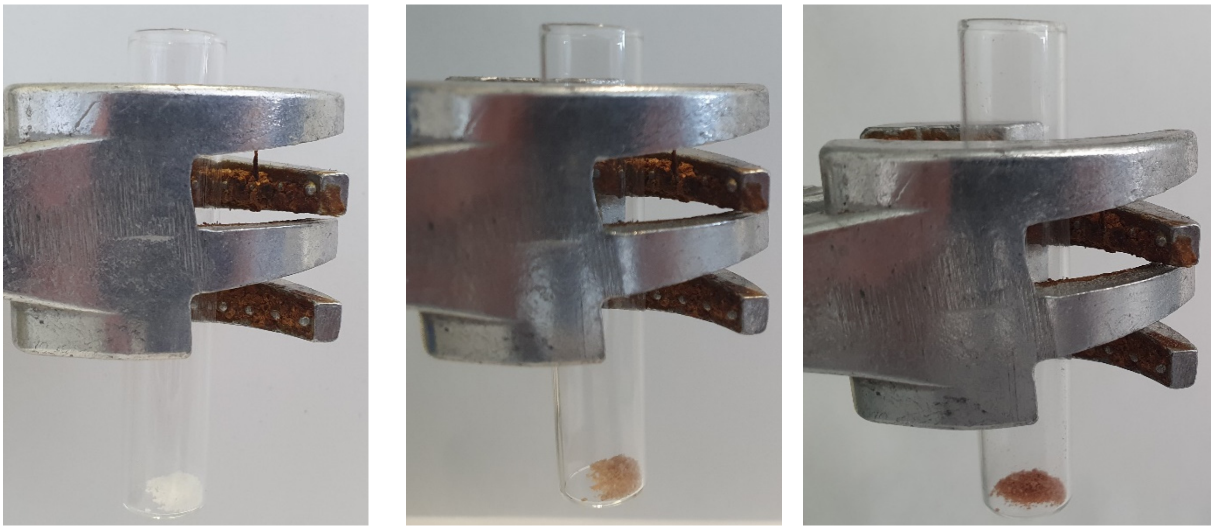

Figure S16. Photographs of the decomposition process of **4** at ambient atmosphere. Left: initial sample, middle: after 3.5 h, right: after 20 h. Increasingly dark color of the solid indicates formation of iodine.

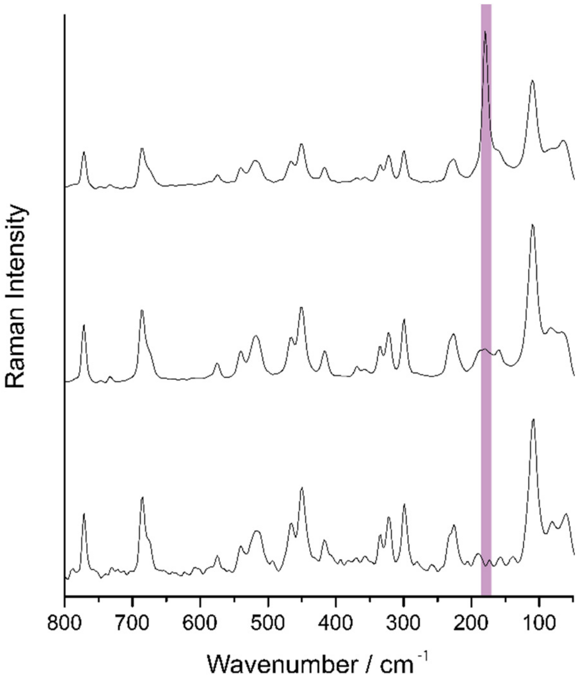

Figure S17. Raman spectra (1064 nm, 298 K) of the decomposition process of **4** at ambient atmosphere. bottom: initial sample, middle: after 3.5 h, top: after 20 h. Purple bar indicates formation of iodine.

Computational Data

NBO analysis:

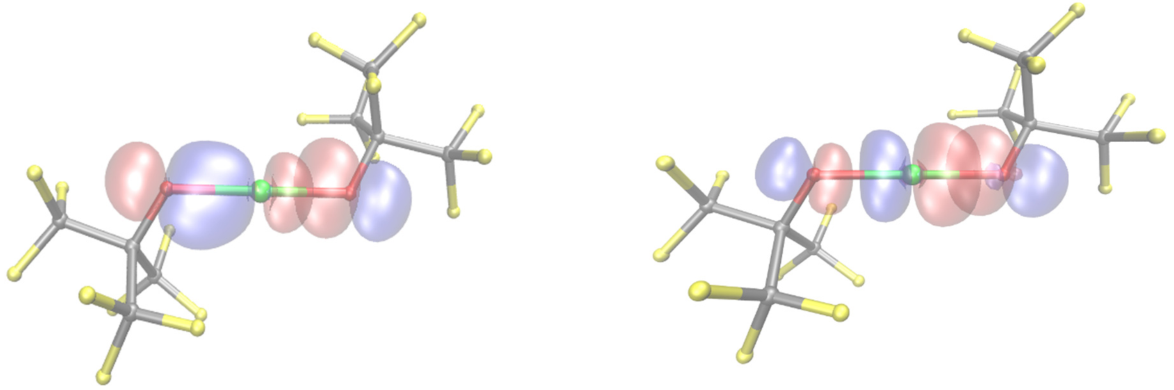

Figure S18. Results from the NBO analysis of $[Cl(OC_4F_9)_2]^-$. Left: NMOs of the O-Cl-O hyperbond. Right: Correlation between the oxygen lone pair and the σ*(Cl-O) NMO.

Figure S18 (left) shows the orbitals taking part in the 3-center-4-elektron bond according to the NBO analysis for $[Cl(OC_4F_9)_2]^-$. The occupancy is 3.9 all three derivatives $[X(OC_4F_9)_2]^-$. Figure S18 (right) shows the correlation between the antibonding σ*(Cl-O) with the lone pair of the opposite oxygen atom. For $[Cl(OC_4F_9)_2]^-$ the occupancy of σ*(Cl-O) which is mainly composed of p-type natural atomic orbitals is 0.38. For $[Br(OC_4F_9)_2]^-$ the occupancy is 0.34 and for $[I(OC_4F_9)_2]^-$ 0.27. The σ*(X-O) orbital is mainly (Cl: 74%, Br: 79%, I 84%) composed of a p-type orbital of X.

NPA charges of neutral precursor species:

Molecule	ClF	$ClOC_4F_9$	$BrOC_4F_9$	IOC_4F_9	$I(OC_4F_9)_3$	IF_3
NPA at X	+0.33	+0.25	+0.34	+0.46	1.65	1.73

Xyz coordinates of $[Br(OC_4F_9)_2]^-$ optimized on B3LYP-D3BJ/def2-TZVPP:

```
29
Energy = -4826.751497174
Br   0.0010693   0.0127201  -0.0066797
F   -2.6646632   1.3074957  -3.2319725
F    3.8301136   1.3671272   3.6630571
F    4.2989331  -0.7649331   1.9857114
F   -2.2653308  -2.4413806  -0.2817040
F    2.3507356   2.7970357   2.9785983
F   -4.2257345  -1.9037999  -1.0239453
F    1.7480359   1.1050283   4.1752269
F   -2.5393345   1.6133139  -1.0996654
O    0.9695350   0.5984656   1.7449338
F   -1.7608547  -1.0656366  -4.1877347
F   -3.2008330  -0.6029086   0.3671572
F    3.2192564   0.5706322  -0.3617402
C   -2.9737767   0.7697044  -2.0372936
O   -0.9694993  -0.5704622  -1.7585113
F   -4.3237096   0.7380659  -1.9557791
F    4.2536542   1.8689460   1.0248017
C    2.5690661   1.4881897   3.1939618
F   -2.3234943  -2.7799184  -3.0033810
F   -3.8333876  -1.3688473  -3.6597706
F    2.5082474  -1.6181824   1.1205938
F    2.3088974   2.4309515   0.2597614
C   -2.3095348  -0.6512139  -1.8815783
```

C 3.0335636 1.3932592 0.6823965
C -3.0106539 -1.4114909 -0.6870993
C 2.3098049 0.6573853 1.8785341
F 2.6181406 -1.2936645 3.2509998
C 2.9479623 -0.7729737 2.0543096
C -2.5662086 -1.4729086 -3.2031881

Xyz coordinates of $[BrF_2(OC_4F_9)_2]^-$ optimized on B3LYP-D3BJ/def2-TZVPP:
31
Energy = -5026.366342161
C -2.8898054 0.7101859 -1.9143302
C -2.3459683 -0.7637351 -1.8044037
C -2.5552561 -1.4814371 -3.1940197
F -3.7874935 -1.2868168 -3.7063569
F -2.4012581 1.3145823 -3.0053567
F -2.5416036 1.4399662 -0.8534549
F -4.2377505 0.7549357 -1.9994901
O -1.0025552 -0.8201842 -1.5862474
Br 0.0008541 0.0005133 -0.0019380
O 1.0031595 0.8220860 1.5829530
C 2.3459069 0.7640554 1.8039404
C 2.8873607 -0.7104342 1.9172203
F 2.3959062 -1.3122503 3.0083044
C -3.1600582 -1.5425255 -0.7031691
F -3.2460791 -0.8209302 0.4240194
F -2.5775859 -2.7009230 -0.4091027
F -4.4256087 -1.8165318 -1.0968965
C 2.5535189 1.4836430 3.1928539
F 1.6716872 1.0471305 4.0942666
F 3.7838674 1.2866902 3.7088592
F 2.3823886 2.8091928 3.0714250
C 3.1635316 1.5399443 0.7032529
F 2.5827217 2.6982430 0.4052315
F 3.2518705 0.8160887 -0.4223977
F 4.4282685 1.8137271 1.0997120
F 4.2351472 -0.7570296 2.0049514
F 2.5400904 -1.4413328 0.8568806
F -1.6770185 -1.0409773 -4.0970719
F -2.3802441 -2.8066770 -3.0753554
F 0.3602261 1.5346641 -1.0661960
F -0.3582202 -1.5338633 1.0619166

Xyz coordinates of $[Cl(OC_4F_9)_2]^-$ optimized on B3LYP-D3BJ/def2-TZVPP:
29
Energy = -2712.843989505
C -3.1555319 0.8068693 -1.6109068
C -2.3003365 -0.5150895 -1.6777069
C -2.6287097 -1.2599176 -3.0279459
F -3.9491589 -1.2967823 -3.3100621
F -3.0866670 1.4722704 -2.7797429
F -2.7085617 1.6288403 -0.6612824
F -4.4661506 0.5796830 -1.3632436
O -0.9757615 -0.2558845 -1.7228807
C -2.7225698 -1.4573525 -0.4812155
F -2.8803713 -0.7698998 0.6593606
F -1.7997570 -2.3966411 -0.2709437
F -3.8957190 -2.0932673 -0.7155734
F -2.0244171 -0.6705724 -4.0633994
F -2.1968449 -2.5319067 -2.9848563
Cl -0.0102346 0.2623125 -0.0786941
O 0.9589950 0.8192657 1.5513204

C 2.2946218 0.6401322 1.6408994
C 2.6703877 -0.8610732 1.9384578
F 2.3090099 -1.1987154 3.1909228
C 2.7544577 1.5263550 2.8610313
F 1.9223431 1.3897213 3.8971357
F 3.9928828 1.2222294 3.3061601
F 2.7626917 2.8277580 2.5246217
C 3.0853262 1.1195947 0.3590327
F 2.5358365 2.2239647 -0.1485940
F 3.0905176 0.1825767 -0.6002120
F 4.3817603 1.4053062 0.6287636
F 3.9971998 -1.1052141 1.8317342
F 2.0447614 -1.6945630 1.1078193

Xyz coordinates of $[I(OC_4F_9)_4]^-$ optimized on B3LYP-D3BJ/def2-TZVPP:
57
Energy = -4803.047330096
I 0.0002818 0.0002934 0.0006938
O 0.0336657 0.0057754 2.1481943
O 2.1473199 -0.0145156 -0.0335196
O -0.0336918 0.0249478 -2.1463905
O -2.1470894 -0.0155321 0.0335331
C 2.9947216 -1.0868966 -0.0405149
C -0.0356127 1.1006438 -2.9896255
C -2.9942989 -1.0881862 0.0304107
C 0.0350578 1.0744567 3.0004351
C -0.0446265 0.4887126 -4.4417994
C -1.3096693 2.0130889 -2.8147947
C 1.2529256 1.9902205 -2.8178365
F -1.2116066 3.1685315 -3.5013141
F -2.4116802 1.3871120 -3.2423191
F -1.4944218 2.3320215 -1.5301228
F -0.3145352 1.4100452 -5.3880128
F 1.1467271 -0.0509860 -4.7367275
F -0.9600502 -0.4744620 -4.5484366
F 1.4225664 2.8589000 -3.8339455
F 1.1754783 2.7081204 -1.6833421
F 2.3467592 1.2393339 -2.7478058
C -4.4442707 -0.4711756 0.0415445
C -2.8249032 -1.9725788 -1.2619608
C -2.8231957 -2.0066628 1.3006903
F -3.5131747 -3.1595354 1.1974260
F -1.5396696 -2.3304028 1.4849660
F -3.2494609 -1.3839603 2.4050865
F -5.3938438 -1.3907418 0.3056737
F -4.5483018 0.4874214 0.9620926
F -4.7364993 0.0760358 -1.1470278
F -3.8424355 -2.8389495 -1.4347846
F -2.7543499 -1.2172013 -2.3526842
F -1.6915247 -2.6925902 -1.1881980
C 4.4447670 -0.4696953 -0.0468079
C 2.8260801 -1.9831217 1.2436842
C 2.8233040 -1.9935091 -1.3192380
F 3.5133141 -3.1473147 -1.2272279
F 1.5397293 -2.3154362 -1.5062006
F 3.2493456 -1.3601228 -2.4176040
F 5.3936835 -1.3857480 -0.3251080
F 4.5465614 0.5007557 -0.9550250
F 4.7399022 0.0619437 1.1481567
F 3.8442087 -2.8505101 1.4078270
F 2.7554926 -1.2379445 2.3414903

F 1.6930411 -2.7029836 1.1637311
C 0.0413365 0.4508600 4.4475575
C 1.3103684 1.9871723 2.8352431
C -1.2524064 1.9664077 2.8335675
F 0.3104926 1.3647873 5.4011958
F -1.1508279 -0.0901962 4.7366212
F 0.9558222 -0.5138675 4.5480505
F 1.2107346 3.1389956 3.5275083
F 2.4104198 1.3580356 3.2635708
F 1.5001154 2.3127651 1.5530840
F -1.4223699 2.8284191 3.8552072
F -1.1726514 2.6917757 1.7039430
F -2.3470545 1.2172483 2.7571895

Xyz coordinates of $[OC_4F_9]^-$ optimized on B3LYP-D3BJ/def2-TZVPP:
14
Energy = -1126.383048342
O 0.1606553 0.1243953 -1.8147659
C 0.0471858 0.0365452 -0.5346221
C 1.4638150 0.1138407 0.2033967
C -0.6306575 -1.3390026 -0.0823163
C -0.8504350 1.2118307 0.0732536
F 2.1545307 -1.0400533 0.0489035
F 2.2244482 1.0860201 -0.3252964
F 1.4232129 0.3505025 1.5459543
F -0.5041534 -1.6585331 1.2376013
F -0.1167314 -2.3724414 -0.7686542
F -1.9597553 -1.3317953 -0.3386093
F -0.1765178 2.3857558 0.0729372
F -1.9513577 1.4051529 -0.6709560
F -1.2842400 1.0277825 1.3531737

Xyz coordinates of $ClOC_4F_9$ optimized on B3LYP-D3BJ/def2-TZVPP:
15
Energy = -1586.409178232
C 0.7394859 0.0431451 1.3047987
F 1.3636821 -1.1401044 1.3086897
C -0.1179315 0.2222003 0.0032407
C 0.7022606 -0.1983228 -1.2713965
F 0.7265311 -1.5286733 -1.4036421
F -0.0424247 0.1131604 2.3865405
F 1.6620711 1.0017873 1.4080856
C -1.4293954 -0.6512033 0.0961645
F -2.0076899 -0.7404558 -1.1044666
O -0.6539266 1.5208972 -0.0835057
Cl 0.3903483 2.7894225 -0.5477707
F -1.1515634 -1.8841156 0.5263841
F -2.3032596 -0.1003982 0.9355823
F 1.9678446 0.2276773 -1.1915098
F 0.1539674 0.3249832 -2.3671945

Xyz coordinates of $BrOC_4F_9$ optimized on B3LYP-D3BJ/def2-TZVPP:
15
Energy = -3700.305358691
C 0.7361939 0.0318509 1.3053324
F 1.3604706 -1.1513770 1.3109693
C -0.1211994 0.2172687 0.0044934
C 0.6988836 -0.2120028 -1.2672873
F 0.7273613 -1.5419986 -1.3974516
F -0.0432753 0.1041231 2.3886638
F 1.6612267 0.9904200 1.4076474

C	-1.4323093	-0.6596866	0.0979541
F	-2.0120646	-0.7472290	-1.1025533
O	-0.6514586	1.5099078	-0.0808484
Br	0.4197796	2.9396219	-0.5759753
F	-1.1539431	-1.8944066	0.5249985
F	-2.3062258	-0.1127821	0.9395004
F	1.9646158	0.2184076	-1.1890526
F	0.1519447	0.3078828	-2.3663909

References

[1] P. Pröhm, J. R. Schmid, K. Sonnenberg, S. Steinhauer, C. J. Schattenberg, R. Müller, M. Kaupp, P. Voßnacker, S. Riedel, *Angew. Chem. Int. Ed.* **2020**, *59*, 16002.
[2] J. R. Schmid, P. Pröhm, P. Voßnacker, G. Thiele, M. Ellwanger, S. Steinhauer, S. Riedel, *Eur. J. Inorg. Chem.* **2020**, *2020*, 4497.
[3] A. R. Mahjoub, K. Seppelt, *Angew. Chem. Int. Ed.* **1991**, *30*, 323.
[4] T. S. Cameron, I. Krossing, J. Passmore, *Inorg. Chem.* **2001**, *40*, 4488.
[5] Y. Yan, J. T. Mague, J. P. Donahue, *CCDC 741550: Experimental Crystal Structure Determination*, Cambridge Crystallographic Data Centre, **2009**.
